# A mixed-methods study on the interplay between AI-assisted dynamic assessment, reading comprehension, and psychological adaptivity

**DOI:** 10.3389/fpsyg.2026.1798029

**Published:** 2026-05-15

**Authors:** Chaofeng Ding, Xiaoyan Li

**Affiliations:** 1 School of Foreign Languages, Pingdingshan University, Pingdingshan, Henan, China; 2 Department of Public Teaching, Qilu Medical University, Zibo, Shandong, China

**Keywords:** academic buoyancy, academic resilience, AI-assisted dynamic assessment, artificial intelligence, dynamic assessment, reading comprehension

## Abstract

Dynamic assessment (DA), grounded in Vygotsky's sociocultural theory (SCT), evaluates learners' developmental potential through mediated support within the zone of proximal development (ZPD). However, limited research has examined how AI-assisted DA (AI-DA) may influence both learners' psychological attributes and language performance. This concurrent mixed-methods study investigated the effects of AI-assisted DA (AI-DA) on Chinese English-as-a-Foreign-Language (EFL) learners' academic buoyancy, academic resilience, and reading comprehension. Conducted at a university in China, the study involved two intact classes (*N* = 92) randomized into an experimental group receiving AI-assisted DA and a control group undergoing teacher-fronted sessions over 16 weeks. Qualitative data were collected through semi-structured interviews and focus group discussions to explore learners' perceptions of buoyancy and resilience, while quantitative data were obtained using a validated reading comprehension test. hematic analysis revealed that AI-DA enhanced learners' academic buoyancy by reducing daily frustrations and strengthened resilience through adaptive scaffolding that encouraged persistence. The quantitative analyses using t-tests and Analysis of Covariance (ANCOVA) demonstrated that the experimental group exhibited significantly higher reading comprehension compared to their peers in the control group. Overall, the findings suggest that AI-assisted dynamic assessment can simultaneously support L2 reading development and learners' psychological adaptability in academic contexts.

## Introduction

Efficient instruction is intrinsically connected to efficient assessment, since each functional evaluation concurrently offers an instructional opportunity (Vygotsky, 1997). Consequently, the integration of evaluation and instruction is vital for promoting language development. DA, rooted in Vygotsky's ZPD, is a methodology that integrates instruction and evaluation into a dialectically cohesive process (Lantolf et al., 2017). Vygotsky's dialectical principle posits that evaluation and instruction are fundamentally interconnected elements of a unified continuum, rendering them virtually inseparable (Lantolf, 2009). This integrated method organizes mediator-learner interactions within the ZPD, facilitating both a detailed assessment and advancement of developmental progress (Poehner, 2018).

Vygotsky (1978) characterized ZPD as the difference between what an individual may achieve autonomously and what can be attained with guided support or mediation. Mediation facilitates the transition of learners from other-regulation to self-regulation of their performance (Aljaafreh and Lantolf, 1994). This process necessitates that learners assimilate suitable mediation forms offered by an informed interlocutor throughout interactions (Lantolf et al., 2017). Mediators and learners participate in a collaborative dynamic, collectively sharing responsibility for different facets of the learning activity (Van Compernolle, 2015). Through the promotion of collaborative involvement, mediators facilitate learners in transcending their existing developmental capabilities, a process attainable via DA but not through conventional assessment methods that overlook the ZPD (Ableeva and Lantolf, 2011; Poehner, 2008).

While the theoretical foundations of DA emphasize the importance of mediation in fostering learner development, recent technological advancements have opened new possibilities for delivering such mediation in more scalable and adaptive ways. In particular, the application of information technology in language teaching and assessment has garnered considerable attention from researchers in recent years (Ahmadi, 2018; Lei et al., 2022; Namaziandost and Rezai, 2024; Shadiev and Yang, 2020; Shadiev et al., 2023). The integration of information technology in language teaching and assessment has garnered considerable attention from researchers (Ahmadi, 2018; Lei et al., 2022; Namaziandost and Rezai, 2024; Shadiev and Yang, 2020; Shadiev et al., 2023). The integration of information technology in language instruction significantly improves the learning experience by offering individualized, interactive, and communicative educational opportunities (Chun et al., 2016; Rahimi and Fathi, 2022; Shatri, 2020). Educators employ information technology to create virtual learning environments that interest students and facilitate language acquisition (Fathi and Rahimi, 2024; Loncar et al., 2023; Nguyen and Le, 2023). AI has emerged as a significant asset in language teaching, enhancing student learning outcomes (Huang et al., 2023; Knox, 2020; Pikhart, 2020).

Within language learning contexts, one of the most important domains where technology-mediated instruction and assessment can play a transformative role is reading comprehension, a foundational skill for academic success. Reading and understanding text is an essential ability vital for academic achievement and lifelong education (National Reading Panel et al., 2000), particularly in online learning, which necessitates supplementary digital competencies. The primary objective of reading is to derive and formulate meaning from various texts (Snow, 2002). Reading comprehension is fundamental to academic advancement, as it supports content-area learning across different fields of study. Reading comprehension necessitates that readers construct a mental representation from the text by integrating both conceptual knowledge (such as vocabulary and metalinguistic awareness) and procedural knowledge (including reading strategies) while engaging with the text (Snow, 2002; Anmarkrud and Bråten, 2009; Daugaard et al., 2017). Some research (Bharuthram, 2012; Afflerbach et al., 2015) reveals that some university students are inadequately equipped to effectively engage with academic texts or experience reading difficulties (Smagorinsky, 2001; Cox et al., 2003), potentially constraining their academic training centered on written materials.

While reading comprehension forms a cornerstone of academic learning, students' ability to thrive in this domain is not solely cognitive but also depends on their psychological resources. From a sociocultural standpoint, mediation within the ZPD supports not only cognitive development but also the internalization of strategies for managing challenges. Through scaffolded interaction, learners gradually develop self-regulation, confidence, and persistence, which underpin psychological resources such as academic buoyancy and resilience. In this way, the ZPD serves as a context for both cognitive and affective growth, linking social mediation to learners' ability to cope with setbacks and sustain effort. In this regard, a construct that has been a focal point in positive psychology and foreign language acquisition is academic buoyancy. It pertains to students' capacity to adeptly navigate their daily, non-severe hurdles and impediments encountered in the academic setting, including low grades, competing deadlines, high-pressure examinations, and rigorous coursework (Martin and Marsh, 2008). In recent years, a growing number of researchers have investigated the role of buoyancy in academic learning and how this construct might be employed to formulate methods for improving academic performance (Putwain et al., 2019). Previous research has demonstrated a correlation between buoyancy and academic success across diverse disciplines and talents (Collie et al., 2024; Colmar et al., 2019; Gao et al., 2025; Guo et al., 2024; Putwain et al., 2024).

Closely related to academic buoyancy, but conceptually broader in scope, is the construct of academic resilience, which concerns learners' capacity to cope with more significant or prolonged academic challenges. Academic resilience is a crucial determinant of students' capacity to adapt to the university environment and aids in mitigating stress risk. It encompasses students' satisfaction in fulfilling all academic obligations, improving their scholarly accomplishments, and promoting appropriate coping mechanisms in the face of academic stress (Pidgeon et al., 2014). Academic resilience explains how pupils overcome major challenges or setbacks that may impede the learning process. This allows individuals to adapt efficiently and fulfill their academic obligations successfully. Resilience is defined as both a capacity and a process enabling an individual to achieve positive adaptation in the face of challenges and adversities (Masten, 2018; Reyes et al., 2018).

Although DA has been widely recognized as a powerful means of integrating instruction and assessment, its application in second language (L2) contexts faces practical limitations. Traditional DA is time-consuming, requires extensive mediator expertise, and is difficult to scale in group-based classroom settings (Lantolf and Poehner, 2004). Recent advances in AI provide new opportunities to overcome these limitations by offering adaptive, individualized, and context-sensitive mediation. Yet, despite the growing interest in AI-driven learning and assessment, little empirical evidence exists regarding how AI-DA can be operationalized in L2 classrooms and what outcomes it can yield. Furthermore, existing DA research has focused primarily on cognitive and linguistic outcomes (Jeon, 2023; Poehner and Wang, 2021; Kao, 2022; Chen et al., 2022), with limited attention to learners' psychological resources. Constructs such as academic buoyancy and academic resilience have emerged as strong predictors of academic success, but their interplay with AI-DA remains underexplored. At the same time, reading comprehension—a core academic skill underpinning success across disciplines—continues to pose challenges for many L2 learners. The lack of research examining the intersection of AI-assisted DA, buoyancy, resilience, and L2 reading comprehension creates a critical gap in the literature. Addressing this gap is essential for understanding not only how technology-enhanced DA influences linguistic performance but also how it supports learners' psychological adaptability in academic contexts.

Although this study examines multiple outcomes, its central focus is to investigate how AI-DA functions as a mediational framework for supporting L2 learning. Within this framework, reading comprehension is treated as the primary learning outcome, while academic buoyancy and academic resilience are examined as complementary psychological resources that may emerge through scaffolded mediation within learners' ZPD. This prioritization reflects the study's broader aim of demonstrating how AI-mediated DA can simultaneously support linguistic development and psychological adaptability. By structuring the investigation around AI-DA as the core pedagogical mechanism, the study seeks to clarify how cognitive and psychological outcomes interact within technology-mediated sociocultural learning environments.

## Literature review

### Theoretical framework

Vygotsky's Sociocultural Theory (SCT) and the ZPD construct provide significant implications for evaluation (Kushki and Nassaji, 2024). SCT-informed assessment approaches, unlike traditional methods that limit evaluation to individual student performance without external aid, incorporate extra resources, including instructor support (Gipps, 2002). This transition alters the dynamics of the teacher-student relationship from a conventional hierarchical framework to a communicative one, where the formerly neutral and impartial teacher adopts a teaching-assisting role, and the student becomes more actively involved in the learning process (Gipps, 2002; Lidz and Gindis, 2003; Taylor et al., 1997). From an SCT perspective, learning and assessment are therefore inseparable processes, as development occurs through mediated interaction within the learner's ZPD.

DA is based on the concept of ZPD (Vygotsky, 1987). It not only emphasizes the past by diagnosing learners' developed abilities, as seen in traditional static assessment, but also considers future growth by prioritizing the enhancement of learners' developing capacities through mediation (Gipps, 2002). Mediation provided to learners during collaborative contact is crucial for future development (Lantolf, 2009). The capabilities of learners today, facilitated by mediation, reflect their potential for independent action tomorrow (Vygotsky, 1978). In this sense, DA operationalizes SCT principles by embedding instructional support directly into the assessment process, transforming evaluation into a developmental activity.

There are two primary types of mediation employed in DA: the interactionist approach and the interventionist approach (Lantolf and Poehner, 2004). In the former, mediation is continuously negotiated and adjusted based on learners' responsiveness during mediator-learner dialogues, whereas in the latter, mediation is pre-constructed and standardized, organized from the most implicit to the most explicit (Lantolf and Poehner, 2011). Considering the respective advantages of the two techniques to DA, they might be amalgamated to formulate effective mediation in educational settings to optimize the impact of mediation on language development (Poehner et al., 2015). However, DA has faced criticism for its conceptual ambiguity, dubious psychometric characteristics, and time-intensive implementation (Grigorenko and Sternberg, 1998), resulting in limited adoption among educational psychologists (Hill, 2015). Consequently, educators and assessment specialists have focused on technology-mediated DA (Poehner et al., 2017). This shift toward technology-mediated mediation has opened new possibilities for scaling DA practices while maintaining the individualized support central to SCT.

DA significantly influences the evaluation of L2 reading in educational environments. It presents a dynamic and interactive methodology for assessment, prioritizing the continuous enhancement of language abilities above a fixed evaluation of proficiency (Kushki and Nassaji, 2024). In the realm of L2 reading, DA enables educators to evaluate a student's current proficiency while also discerning their learning potential and the elements affecting their language growth. This viewpoint promotes a tailored and focused instructional strategy by pinpointing distinct strengths and regions requiring more assistance (Kushki and Nassaji, 2024). By acknowledging the fluidity of language acquisition, educators can customize their instructional and evaluative approaches to more effectively address individual learner requirements, so cultivating a more efficient and inclusive educational atmosphere for L2 readers in the classroom.

### AI in education

AI is a rapidly advancing field within computer science that aims to develop systems capable of performing tasks that typically require human intelligence (Terra et al., 2023). These activities demonstrate human cognitive processes, including learning, thinking, problem-solving, pattern recognition, and prediction (Siemens et al., 2022). AI implementations may manifest in several modalities, utilizing either physical or virtual components, and can operate within autonomous or decentralized frameworks. Moreover, the implementations can manifest as perceptive, autonomous creatures capable of interacting with their environment and exercising discernment (Luxton, 2016). AI can be categorized into two types: narrow or weak AI, which is designed for certain tasks, and general or strong AI, which possesses the ability to do intellectual tasks at a level comparable to that of humans (Kay, 2012). Recent investigations suggest that the implementation of AI in education positively influences language acquisition (Kohnke et al., 2023), offering interactive learning opportunities (Chiu et al., 2024). From a sociocultural perspective, AI-based tools can function as mediational artifacts that support learners' engagement with tasks and facilitate scaffolded interaction. The implementation of AI in language acquisition may have both advantageous and detrimental consequences for educators, who are central to their students' education and facilitate their learning incrementally.

Many studies have investigated the influence of AI-enhanced language learning aids on the academic performance of English language learners (Divekar et al., 2022; Fitria, 2023). Zheng and colleagues (Zheng et al., 2023) conducted a meta-analysis on the impact of AI on learning outcomes and perceptions. Upon analyzing 24 studies with 2,908 participants from 2001 to 2020, it was shown that AI exerted a more significant influence on learning outcomes than on learners' perceptions. This indicates that the majority of research has concentrated on the beneficial effects of AI on academic performance, while learner perceptions have been a secondary concern. In another study, Xu and colleagues (Xu et al., 2023) examined the impact of AI-assisted language learning on the speaking and interpersonal abilities of English learners. Their research demonstrated that the utilization of AI tools equipped with speech recognition capabilities improved learners' language proficiency and actively involved them in interactive language learning activities. Two recent studies illustrate AI's mediational affordances in providing contingent scaffolding. First, Kim (2026) fine-tuned a GPT-based chatbot to provide graduated mediation for beginner learners of Korean, adjusting its feedback contingent on learner responses. Qualitative analysis of 280 learner-chatbot interactions identified conversational, instructional, and developmental mediation types, while quantitative measures showed gains in sentence length and lexical diversity. This study demonstrates that AI can emulate core principles of Vygotskian mediation, supporting learner progression from actual development toward the ZPD. Second, a study with Iranian EFL learners compared AI-assisted DA, interventionist DA, and a control condition (Kargar Behbahani et al., 2026). The AI-assisted DA group outperformed peers on receptive and productive vocabulary measures, highlighting AI's ability to function as a co-mediator, providing tailored scaffolding that enhances both learning outcomes and engagement. Despite these advances, relatively little research has examined how AI might support mediation processes central to DA, particularly in ways that simultaneously promote cognitive development and learners' adaptive psychological responses to academic challenges.

### Academic buoyancy

Academic buoyancy, defined as a student's ability to effectively manage and recover from typical daily academic problems such as subpar results or deadline stress, is gaining recognition in educational psychology for its role in promoting student performance, motivation, and well-being (Martin and Marsh, 2008; Martin, 2013). Importantly, it contrasts with academic resilience: buoyancy pertains to everyday pressures, rendering it particularly pertinent for the majority of students, whereas resilience pertains to reactions to substantial hardship (Martin, 2013; Datu and Yang, 2021; Putwain et al., 2020). This distinction underscores the many coping techniques involved. Martin and Marsh (2008) characterized academic buoyancy as the ability to “bounce back” after normal setbacks, distinguishing it from more comprehensive resilience theories (Putwain et al., 2023). Efficient handling of these daily stresses is essential for students' ongoing academic engagement and stable performance (Collie et al., 2015; Putwain et al., 2022). Resilient students generally proficiently navigate these challenges, rebound from transient performance declines, and sustain an optimistic learning attitude (Miller et al., 2013; Mohammad Hosseini et al., 2024).

Studies generally associate academic buoyancy with various favorable educational outcomes. Buoyant students have enhanced engagement and motivation while confronting academic problems (Bostwick et al., 2022; Liu et al., 2025; Thomas and Allen, 2021), and elevated buoyancy in high school forecasts continued accomplishment, even in the face of adversity (Collie et al., 2015). This capability also enhances agency and self-efficacy, as resilient students frequently experience increased control over their academic paths (Collie et al., 2017). Moreover, academic buoyancy correlates with increased positive achievement-related feelings and less negative emotional reactions to obstacles, hence enhancing performance (Putwain et al., 2022). A positive correlation between motivation and involvement has been documented across several cultural contexts, particularly among Filipino students (Datu and Yang, 2021). In addition to academic success, buoyancy is substantially associated with student well-being; for example, more buoyant primary school pupils exhibit enhanced general well-being, improved emotional control, and less stress (Miller et al., 2013). Within technology-mediated learning environments, scaffolded support and immediate feedback may play a crucial role in sustaining learners' buoyancy by helping them manage everyday academic setbacks during complex tasks such as reading comprehension.

### Academic resilience

A meta-analytic study on resilience offers multiple definitions that broadly define resilience as an individual's capacity to maintain optimal performance in adverse settings and to swiftly recuperate from stressors (Hartmann et al., 2020). Luthar and colleagues (Luthar et al., 2000) characterize resilience as a dynamic process involving positive adaptation amidst considerable adversity, linking this personal trait to the capacity to leverage both contextual and personal resources to endure hardships, adjust to change, exhibit flexibility, undergo positive behavioral transformation in challenging circumstances, and achieve positive adaptation in adverse contexts. Luthans (2002) defines resilience as a cultivable positive psychological capacity to recover from adversity, uncertainty, conflict, failure, or even positive change, progress, and heightened responsibility. Masten (2014) asserts that resilience is fundamentally the ability of a dynamic system to effectively adjust to disruptions that jeopardize its function, viability, or development. In the academic setting, resilience enables students to surmount adversities, enhance well-being, attain academic success, and boost employment prospects (Ang et al., 2021).

Resilience is particularly crucial for mental health studies among young demographics, such as college students (Tarriño-Concejero et al., 2024). Students with good academic resilience frequently attain more achievement and encounter fewer psychological challenges (Collie et al., 2017). Academic resilience is essential for sustaining learning dedication in the face of setbacks (Hirvonen et al., 2020). It is a crucial indicator of successful aging and prospective professional achievement (Reich et al., 2020). Theory of accomplishment motivation posits that motivation is affected by anticipated success and the worth of the job (Atkinson, 1957). Academic resilience, based on this notion, denotes a student's ability to maintain motivation and engagement in the learning process (Pitzer and Skinner, 2017). Thorough study on college students is crucial for recognizing challenges and formulating support solutions to improve achievement, retention, and well-being. Elevated academic resilience enables pupils to effectively navigate challenges, utilize constructive techniques, and mitigate anxiety (Grayson, 2023). Nonetheless, the academic endurance of college students is frequently neglected. It denotes the capacity to surmount challenges and adverse experiences in the learning process (Martin and Marsh, 2008). Due to the distinct requirements of higher education, further investigation is necessary about the correlation between resilience, student well-being, and determination in achieving academic objectives (Shannon, 2020). In instructional contexts where learners receive adaptive support and guidance, such as mediated learning environments, resilience may be strengthened through repeated opportunities to confront and overcome academic difficulties.

### Interplay between DA and academic outcomes

Evaluations of reading and associated skills that gauge acquired knowledge may hinder the forecasting of future reading proficiency. Static assessments frequently lead to floor effects during the initial phases of reading instruction and may serve as particularly unreliable predictors for children from culturally and linguistically diverse (CLD) backgrounds. DA emphasizes learning potential by evaluating responses to instruction, potentially rendering it a less biased assessment method. Accordingly, DA has been increasingly explored as a framework capable of linking assessment practices with learners' developmental trajectories. Dixon et al. (2023) performed a comprehensive evaluation of the literature to evaluate the efficacy of dynamic measures of reading and associated skills in predicting variations in the development of children's reading abilities across time. Seventeen peer-reviewed papers fulfilled the inclusion criteria, encompassing 18 investigations published from 1992 to 2020. Once static predictors were considered, dynamic measures of phonological awareness and decoding elucidated a substantial proportion of variance in the development of word reading accuracy (1–21%) and word reading fluency (generally 1–9%), whereas variance in reading comprehension outcomes was elucidated by dynamic measures of morphological awareness (4–33.4%) and a singular dynamic decoding assessment (1%). A solitary paired-associate nonword learning exercise accounted for 6% of the unique variable in subsequent nonword reading accuracy and fluency. The findings indicated that DA can access variation not accounted for by conventional static measures; however, no research has specifically investigated the validity of DA for children from CLD backgrounds.

Bashiri and Ebadi (2025) investigated the pragmatic competence of EFL learners and their perspectives after participating in game-based group DA (GDA). Sixty upper-intermediate learners were randomly allocated to experimental and control groups. All groups received instructions through a digital game titled Trace Effects. The experimental groups engaged in a cumulative GDA course with teacher mediation, while the control group did not get synchronous feedback. Alongside the identification of a typology of mediational motions for the experimental group by microgenetic analysis, ANCOVA was performed to ascertain potential differences between the control and experimental groups. The findings indicated that the experimental group enhanced their pragmalinguistic and sociopragmatic abilities, reflecting their microgenetic development.

DA, a novel evaluative method, has garnered interest as a conceptual framework that highlights the socially interactive aspect of learning. Despite receiving attention in language testing and evaluation, the viability of DA in engaging bigger cohorts raises concerns. The deficiency of DA necessitates the implementation of GDA. Kao (2022) investigated the degree to which mediation offered through GDA frameworks—simultaneous and cumulative—facilitated the literacy development of a cohort of language learners. This study examines the rhetorical awareness of five intermediate L2 Chinese learners by their performance on reading and writing assignments in Chinese. The ‘*Qi-cheng-zhuan-he*' rhetorical structure was chosen as it is regarded as the most challenging aspect for Chinese learners. Findings indicated: (1) the mediation offered to participants via both concurrent and cumulative GDA methodologies enhanced their comprehension of the ‘*Qi-cheng-zhuan-he*' approach, (2) the greater the frequency of a participant's engagement as the primary interactant, the superior the learning outcomes exhibited. Each individual participant exhibited distinct developmental levels, resulting in varying degrees of receptivity to the teacher's mediation; yet, their active engagement, whether through vocal or nonverbal behaviors, would enhance their learning performance.

In a ground-breaking study, Jeon (2023) examined the impact of Chatbot-Assisted DA (CA-DA) on vocabulary acquisition and offered insights about learner capabilities derived from its application. Bashiri and Ebadi (2025) employed mediating chatbots to deliver DA to several learners concurrently, offering each learner a human-like contact. The chatbots were developed on Google's Dialogflow. Fifty-three Korean EFL primary school students, exhibiting comparable vocabulary sizes, participated in this study. Participants were randomly allocated to three groups: CA-DA, Chatbot-Assisted Non-Dynamic Assessment (CA-NDA), and a control group. During two therapy sessions, the participants were instructed to read texts and ascertain the meanings of underlined target terms. The chatbots offered graded support to learners in the CA-DA group and only focused on word definitions for learners in the CA-NDA group. The control group did not employ chatbots. Two posttests (receptive and productive) were conducted immediately following and 2 weeks subsequent to the second treatment session. Records of interactions between the chatbots and learners during the two therapy sessions were also gathered. The posttest results indicated that vocabulary improvements in the CA-DA group were markedly superior than those in the other groups. The examination of interactions between chatbots and learners throughout the CA-DA sessions yielded comprehensive evidence of learner progression. The findings indicate that CA-DA may facilitate vocabulary acquisition while also providing diagnostic insights on individual learners' vocabulary development.

Taken together, these studies suggest that DA has consistently demonstrated its effectiveness in supporting linguistic development, yet its integration with emerging technologies remains limited.

Despite the growing body of research on DA and its role in mediating L2 learning, the incorporation of AI into this process remains strikingly underexplored. Except for Jeon's pioneering study (Jeon, 2023), no empirical research has systematically examined how AI may operate as a co-mediator alongside the teacher to provide graduated, contingent support in DA. This is a notable oversight, given that AI-driven tools are increasingly being integrated into educational contexts and have the potential to augment human mediation in powerful ways. Furthermore, while prior research on DA has largely centered on linguistic gains such as vocabulary or grammar, little attention has been devoted to investigating how AI-assisted DA might shape broader and equally critical dimensions of learner development, such as reading comprehension, academic buoyancy, and academic resilience. These constructs, which speak to learners' capacity to process texts effectively and to withstand challenges in their academic journeys, are crucial in today's complex and demanding learning environments. Addressing this gap requires an integrative perspective that connects sociocultural mediation, AI-assisted learning, and psychological adaptability within a unified framework. The lack of systematic research into the intersection of AI, DA, and these underexplored learner outcomes leaves a substantial gap in the literature—one that this study seeks to address. Therefore, the following research questions are addressed in this study:

1. Does AI-DA contribute to L2 reading comprehension?
2. How do L2 learners exposed to AI-DA experience and perceive academic buoyancy?
3. How does AI-DA contribute to L2 learners' experience and perception of academic resilience?

## Method

### Design

To explore the interplay between AI-DA and Chinese EFL learners' academic buoyancy, academic resilience, and reading comprehension, the present study employed a concurrent mixed-methods design. This design allowed the researchers to collect and analyze quantitative and qualitative data simultaneously, capturing both the measurable outcomes of AI-DA and learners' experiences during the intervention. Simultaneous data collection was particularly important because the research aimed to link learners' performance outcomes directly with their socio-emotional experiences; a sequential design could have delayed or fragmented this connection, potentially obscuring how AI-DA influenced cognitive and affective dimensions in real time. This design was deemed appropriate because it allowed for the simultaneous collection and analysis of quantitative and qualitative data, thereby capturing both the measurable outcomes of AI-DA and the nuanced learner experiences underpinning those outcomes. While the quantitative strand enabled the examination of the statistical significance of AI-DA effects, the qualitative strand provided rich, contextualized insights into how learners perceived and responded to AI-assisted mediation. By integrating these complementary data sources, the study aimed to offer a more holistic understanding of the extent to which AI-DA can support not only learners' cognitive performance but also their socio-emotional adaptability in academic contexts.

### Context and participants

The study was conducted at a university in China and involved two intact English classes comprising a total of 92 learners. To ensure comparability, the classes were randomly assigned to either the experimental group (*n* = 46), which received AI-DA, or the control group (*n* = 46), which participated in traditional teacher-fronted instruction. The intervention spanned a full academic semester of 16 weeks. Both classes had a balanced gender distribution, with equal numbers of male and female students, which minimized the potential for gender-related bias. Participants' ages ranged from 18 to 27 years, reflecting the typical demographic of undergraduate students in Chinese higher education. All participants were native speakers of Chinese, monolingual in their first language, and none had any prior experience traveling to or residing in English-speaking countries. This linguistic and cultural homogeneity reduced the possibility of extraneous factors influencing their English learning. Proficiency levels were established using the Oxford Quick Placement Test, which showed that all learners were at an A2 level of English proficiency on the CEFR scale, ensuring a relatively uniform starting point. Moreover, none of the participants reported studying any foreign language other than English, which further controlled for external language-learning effects. The use of intact classes, random assignment, and careful profiling of participants enhanced the ecological validity of the study while maintaining a reasonable degree of experimental control.

### Instruments

Data for the study were collected through both qualitative and quantitative instruments to capture the multifaceted impact of AI-DA. For the qualitative strand, semi-structured interviews and a focus group discussion were employed to investigate how AI-DA contributed to learners' academic buoyancy and academic resilience. The qualitative data was gathered in Chinese and the participants' responses were translated into English. To ensure the accuracy of the translation, it was checked and confirmed by two PhD holders in Applied Linguistics. The interview protocol was designed to elicit participants' perceptions of the role of AI as a co-mediator, their coping strategies in response to academic challenges, and their reflections on the learning experience. To establish the credibility of the qualitative data, two specialists in applied linguistics reviewed the instruments for clarity, relevance, and alignment with the study objectives. Pilot interviews were also conducted to refine the structure and sequencing of the questions. Dependability was further ensured by employing systematic coding procedures, triangulating data across interviews and focus group discussions, and conducting member checks, where participants were invited to verify the accuracy of the interpretations of their responses.

To go in further detail, semi-structured interviews were conducted with a subsample of participants from both the experimental and control groups to explore how AI-DA influenced their academic buoyancy and academic resilience. The interviews focused on learners' perceptions of AI as a co-mediator, their strategies for overcoming daily academic challenges, and the extent to which the intervention supported their socio-emotional adaptability. An interview guide was prepared in advance, but participants were encouraged to elaborate freely, allowing the interviewer to probe emerging themes. Each interview lasted between 20 and 30 min and was audio-recorded with participants' consent. The credibility of the interview data was established through expert review of the protocol by two applied linguistics specialists, who confirmed alignment with the study's objectives. Dependability was further strengthened by piloting the interview with a small group of learners, refining the questions based on their feedback, and employing member checks to verify the accuracy of interpretations. Guiding questions for the interview included:

1. How do you usually cope with challenges such as low grades, heavy coursework, or exam stress?
2. What role, if any, did the AI system play in helping you deal with these challenges?
3. How did AI-assisted feedback compare with traditional teacher feedback?
4. Did working with AI mediation change your confidence or strategies for reading comprehension tasks?
5. In what ways, if any, did you feel more capable of overcoming academic challenges after using AI-DA?

To complement the individual interviews and facilitate collective reflection, a focus group discussion was held with 8 participants from the experimental group after the treatment phase. The focus group aimed to elicit shared experiences of how AI-DA shaped learners' buoyancy, resilience, and engagement with reading comprehension tasks. The group setting allowed participants to interact with each other, exchange perspectives, and build on one another's responses, providing richer and more diverse data than individual interviews alone. The session lasted approximately 45 min and was moderated by the researcher, who ensured balanced participation and encouraged both positive and critical perspectives. All discussions were audio-recorded, transcribed verbatim, and anonymized for analysis. To ensure credibility and dependability, the discussion guide was reviewed by experts, and triangulation with interview data was performed. The guiding questions for the focus group discussion included:

1. How did the AI-assisted assessment sessions differ from the teacher-fronted sessions you experienced before?
2. Did the AI mediation help you feel more resilient when facing difficult reading comprehension tasks? How?
3. In what ways did the AI system encourage or discourage your participation and persistence?
4. Can you describe any changes in how you deal with setbacks or academic pressure since using AI-DA?
5. As a group, do you think AI can play an effective role as a co-mediator in future English classes? Why or why not?

For the quantitative strand, a reliable, content- and construct-validated reading comprehension test adapted from Namaziandost et al. (2023) was employed to examine how AI-DA influenced learners' cognitive outcomes. The test comprised 20 objective items in multiple-choice, true/false, and fill-in-the-blank formats, designed to assess learners' comprehension and interpretation of written texts. To measure learning gains, the test was administered twice: once as a pretest prior to the treatment and once as a posttest following the intervention. The same instrument was used for both administrations to ensure comparability and consistency of measurement, thereby minimizing the risk of introducing construct-irrelevant variance across testing occasions. To reduce the potential threat of test familiarity or practice effects, a 16-week interval between administrations provided sufficient time to diminish memory-based responses, ensuring that observed improvements could be attributed to the instructional treatment rather than test repetition. Taken together, these instruments ensured that the study systematically addressed both the cognitive and socio-emotional dimensions of learners' development in response to AI-DA.

### Ethical considerations

The study strictly adhered to ethical research guidelines to protect the rights and well-being of participants. All students were informed about the aims, procedures, and potential benefits of the study before data collection began. Participation was entirely voluntary, and students were explicitly told that they could withdraw from the study at any stage without academic penalty. Written informed consent was obtained from all participants prior to their involvement in interviews, focus groups, and tests. To preserve confidentiality, pseudonyms were used in transcriptions and reports, and any identifying information was removed. Audio recordings and transcripts were stored securely and were accessible only to the research team. Anonymity was ensured by reporting findings at the group rather than the individual level.

### Treatment

The instructional treatment lasted 16 weeks and was designed to compare the effects of AI-DA with those of traditional teacher-fronted instruction on Chinese EFL learners' reading comprehension, academic buoyancy, and academic resilience. Both groups were exposed to the same reading comprehension passages, drawn from authentic academic texts (Active 1 textbook) appropriate for learners at the A2 proficiency level as determined by the Oxford Quick Placement Test. Care was taken to ensure that the groups received equal instructional time, identical content exposure, and the same number of practice opportunities, thereby allowing for a fair comparison of the mode of mediation as the key variable.

Learners in the experimental group (*N* = 46) engaged with ChatGPT-4o, which was employed as a co-mediator to deliver mediation. The AI system was prompted to offer graduated and adaptive mediation within the framework of Vygotsky's ZPD. The process followed a consistent cycle:

1. Initial Attempt: Students first attempted the reading comprehension questions independently.
2. Trigger for Mediation: If a learner made an incorrect attempt or expressed difficulty, the AI system intervened with scaffolding tailored to the learner's responses.
3. Graduated Mediation: The AI provided support along a continuum, beginning with the most implicit prompts and progressing to more explicit ones if the learner still struggled. Examples included:
  1. ° *Implicit prompt*: “Look again at the sentence containing the word *although*. How does this word affect the meaning of the sentence?”
  2. ° *Contextual strategy*: “Try using the sentences before and after this one to infer the main idea.”
  3. ° *Guided options*: “Do you think the word ‘*allocate'* here means (a) to give out, (b) to explain, or (c) to deny?”
  4. ° *Explicit mediation*: “The word ‘*allocate'* means ‘to give out or distribute.' Can you now explain the sentence in your own words?”

This dynamic cycle aimed not only to improve immediate comprehension but also to encourage learners to adopt self-regulation strategies. By providing constructive hints, prompting metacognitive reflection, and reinforcing persistence, the AI system played a dual role of enhancing academic buoyancy (coping with minor setbacks) and academic resilience (sustained adaptation to challenges over time).

The adaptive and individualized nature of ChatGPT-4o's mediation was a key feature of the experimental condition. Unlike conventional assessment, where incorrect responses are often treated as failures, AI-DA treated them as opportunities for growth, guiding learners through their difficulties and documenting microgenetic development. To ensure fidelity of implementation, the instructor systematically monitored AI interactions by reviewing logs of each student session weekly, verifying that the AI consistently followed the graduated scaffolding sequence, and documenting any deviations from the mediation protocol. This process ensured that all participants in the experimental group received comparable and consistent AI-DA support throughout the intervention.

Learners in the control group (*N* = 46) engaged in teacher-fronted sessions using the same reading comprehension materials. Instruction was delivered in a more traditional, static format, with the teacher explaining difficult vocabulary, summarizing passages, and providing general feedback to the class as a whole. For instance, when a student encountered difficulty with a passage, the teacher typically clarified the meaning directly:

- *Example*: “The author contrasts two perspectives here. The first view is optimistic, but the second is more critical.”

While this approach provided accurate information, it did not involve graduated scaffolding tailored to individual learners' needs. Feedback was collective and relatively uniform, often resolving difficulties for the group at once rather than engaging with each learner's specific zone of development. Thus, unlike the experimental group, the control condition reflected a one-size-fits-all approach, aligning with the logic of static assessment.

The contrast between the two treatments allowed for the isolation of the mediational variable. Both groups:

- Used the same reading passages and tasks.
- Had the same instructional time and frequency.
- Completed comprehension checks through identical formats.

The only systematic difference lay in the mode of mediation: adaptive AI-assisted scaffolding vs. teacher-fronted explanation. This design permitted an evaluation of whether AI-DA not only improved reading comprehension performance but also nurtured learners' academic buoyancy and resilience by modeling persistence, strategy use, and adaptive problem-solving.

### Data analysis procedures

The study employed a concurrent mixed-methods approach, necessitating distinct analytical procedures for the qualitative and quantitative data. Qualitative data from the semi-structured interviews and focus group discussions were analyzed using thematic analysis to identify patterns and themes related to learners' academic buoyancy, resilience, and experiences with AI-DA. All audio-recorded sessions were transcribed verbatim, and the transcripts were read multiple times to ensure familiarity with the data. Coding followed a systematic three-stage procedure. Open coding involved labeling meaningful segments of the transcripts line by line to capture significant ideas and experiences. During axial coding, the initial codes were examined for relationships and grouped into categories, allowing the exploration of how subcategories interacted and contributed to broader concepts. Finally, selective coding was conducted to integrate categories around central phenomena, such as learners' engagement with AI mediation, the development of adaptive strategies, and the cultivation of academic buoyancy and resilience. To enhance credibility and dependability, two researchers independently coded the data, resolving discrepancies through discussion. Member checking was conducted by providing participants with summaries of interpretations for verification, and detailed audit trails documented all analytic decisions.

Quantitative data, obtained from the reading comprehension test, were analyzed using *t*-tests to determine the contributions of AI-DA to reading comprehension. Furthermore, an ANCOVA was used to evaluate the effect of AI-DA on learners' posttest performance while controlling for pretest scores. ANCOVA was selected because it adjusts for baseline differences, allowing the posttest outcomes to more accurately reflect treatment effects. Prior to analysis, data were screened for assumptions, including normality, linearity, homogeneity of regression slopes, and homogeneity of variance. Effect sizes were calculated to determine the magnitude of the AI-DA intervention. Statistical analyses were performed using SPSS version 20, and the results were interpreted in conjunction with qualitative insights to provide a comprehensive understanding of the interplay between AI-DA, academic buoyancy, resilience, and L2 reading comprehension.

The study was designed with replicability in mind, providing detailed documentation of participants, instruments, procedures, and data analysis. The intervention clearly specifies the use of ChatGPT-4o as a co-mediator, including the graduated scaffolding sequence, prompts, and monitoring procedures to ensure fidelity. Qualitative procedures were systematically outlined with guiding questions, duration, and transcription/translation protocols. Quantitative measures were explicitly described. Data analysis methods for both qualitative and quantitative strands are fully reported, ensuring that future researchers can replicate the study and evaluate the effects of AI-DA under comparable conditions.

## Results

### Intersection of AI-DA and academic buoyancy

The qualitative analysis of semi-structured interviews and focus group discussions revealed several insights into how AI-DA contributed to the development of learners' academic buoyancy. Three main themes emerged from the data: (1) timely and adaptive support reduced stress and uncertainty, (2) personalized scaffolding encouraged persistence, and (3) enhanced confidence promoted a positive mindset toward challenges.

The first theme, *timely and adaptive support reduced stress and uncertainty*, reflected how AI-DA provided learners with immediate, context-sensitive feedback, which helped them manage setbacks without becoming overwhelmed. Several participants noted that having access to real-time guidance from ChatGPT-4o allowed them to regain composure when facing difficulties. For instance, one learner explained:

“*When I got stuck on a reading question, the AI didn't just give me the answer but asked me to think about the key words. That made me feel less nervous, like I had a partner guiding me step by step.”*

Similarly, another student commented:

“*Before, I would panic if I didn't understand a text. With the AI, I could calm down because I knew I would get some hints to push me forward.”*

The second theme, *personalized scaffolding encouraged persistence*, highlighted how AI-DA adjusted its mediation to match learners' needs, fostering resilience in the face of academic challenges. Many students emphasized that the individualized prompts motivated them to keep working rather than give up. As one participant noted:

“*The AI kept asking me questions until I figured it out. It felt like it believed I could do it, so I didn't want to quit.”*

Another student elaborated:

“*Sometimes the teacher just moves on if you don't answer, but the AI kept giving me different clues. That made me keep trying until I solved it.”*

This type of tailored support created a sense of persistence, reinforcing learners' ability to cope with temporary setbacks.

The third theme, *enhanced confidence promoted a positive mindset toward challenges*, demonstrated how AI-DA fostered learners' belief in their ability to succeed. Several participants reported that overcoming reading comprehension difficulties through AI-mediated scaffolding improved their confidence, which in turn contributed to buoyancy. One learner stated:

“*I felt stronger after solving difficult passages with the AI's help. Next time I saw a hard text, I thought: maybe I can handle this.”*

Another participant explained:

“*The AI helped me see mistakes as a normal part of learning. I became less afraid of failure, which made me more positive in class.”*

These reflections illustrate how the AI mediation not only improved immediate performance but also nurtured broader emotional resilience and adaptability.

Taken together, the findings indicate that AI-DA supported learners' academic buoyancy by reducing stress through timely support, encouraging persistence with personalized scaffolding, and enhancing confidence to face future challenges. These outcomes suggest that AI mediation can serve as a co-regulator of affective and cognitive resources, enabling learners to navigate academic obstacles with greater adaptability and optimism.

### Interplay between AI-DA and academic resilience

Thematic analysis of interview and focus group data further revealed how AI-DA contributed to the strengthening of learners' academic resilience. Three salient themes emerged: (1) AI mediation fostered adaptive coping strategies, (2) constructive struggle nurtured long-term perseverance, and (3) reflective learning promoted sustained motivation.

The first theme, *AI mediation fostered adaptive coping strategies*, underscored how learners used the AI's scaffolding to manage academic adversity productively. Instead of avoiding difficult reading tasks, participants reported learning how to break down problems into smaller steps with the guidance of ChatGPT-4o. As one student remarked:

“*When the text was too hard, the AI suggested looking for signal words or breaking the sentence into smaller parts. That strategy stayed with me even after the session.”*

Another learner added:

“*I used to get stuck and wait for someone to give me the answer, but the AI taught me how to search for clues in the text. Now I can try on my own first.”*

These experiences show how AI-DA equipped students with coping mechanisms that extended beyond immediate tasks.

The second theme, *constructive struggle nurtured long-term perseverance*, reflected how learners perceived AI's stepwise mediation as a safe environment for failing, trying again, and eventually succeeding. Unlike traditional classroom settings where mistakes might be stigmatized, the AI system created an opportunity to engage in productive struggle. A participant explained:

“*The AI never got tired of giving me hints, even if I failed many times. It made me realize that struggling is part of the process.”*

Another learner highlighted:

“*I used to lose motivation when I failed, but with the AI, each failure felt like a step closer to understanding. That gave me strength not to give up.”*

This gradual, mediated struggle was seen as essential for building resilience.

The third theme, *reflective learning promoted sustained motivation*, illustrated how AI-DA encouraged students to reflect on their learning progress and reframe setbacks as opportunities for growth. By highlighting learners' incremental improvements, the AI system reinforced a resilient mindset. One learner reflected:

“*The AI reminded me of how I improved compared to last week. That helped me see that difficulties are temporary.”*

Similarly, another participant noted:

“*When I failed, the AI asked me why I thought the answer was wrong. This reflection helped me see mistakes not as failure but as learning moments.”*

Such reflective practices fostered a durable sense of resilience, enabling learners to sustain motivation in the face of future challenges.

Overall, the findings indicate that AI-DA enhanced academic resilience by equipping learners with adaptive coping strategies, providing a supportive space for constructive struggle, and encouraging reflective practices that sustained motivation. These outcomes suggest that AI can function as a co-mediator of resilience, empowering learners to withstand and adapt to academic challenges over time.

### Contributions of AI-DA to L2 reading comprehension

To ensure how AI-DA contributes to L2 reading comprehension, *t*-tests and ANCOVAs were needed. Before their conduct, a Kolmogorov-Smirnov Test determined the degree to which data was normally distributed.

Table 1 demonstrates that the normality assumption was met pre- and post-instruction (*p* >0.05).

**Table 1:** One-sample Kolmogorov-Smirnov Test.

		Pretest scores	Posttest scores
N		92	92
Normal parameters	Mean	4.9022	12.0978
	Std. deviation	1.69070	3.43242
Most extreme differences	Absolute	0.133	0.108
	Positive	0.127	0.098
	Negative	−0.133	−0.108
Kolmogorov-Smirnov Z		1.278	1.038
Asymp. sig. (2-tailed)		0.076	0.232

Table 2 demonstrates that both AI-DA group (*M* = 5.000, *SD* = 1.549), and the control group (*M* = 4.804, *SD* = 1.833) performed similarly pre-instruction.

**Table 2:** Pre-instruction group statistics.

	Condition	*N*	Mean	Std. deviation	Std. error mean
Pretest scores	AI-DA	46	5.0000	1.54919	0.22842
	Control	46	4.8043	1.83327	0.27030

As Table 3 demonstrates, the difference between the AI-DA and non-AI-DA group was insignificant on the pretest (*t* =0.553, *df* = 90, *p* >0.05).

**Table 3:** Pre-instruction independent samples test.

		Levene's test for equality of variances			*t-*test for equality of means					
		*F*	Sig.	*t*	df	Sig. (2-tailed)	Mean difference	Sth. error difference	95% confidence interval of the difference	
									Lower	Upper
Pretest Scores	Equal variances assumed	1.692	0.197	0.553	90	0.582	0.19565	0.35389	−0.50741	0.89871
	Equal variances not assumed			0.553	87.564	0.582	0.19565	0.35389	−0.50767	0.89898

Table 4 reveals the supremacy of the AI-DA condition (M = 14.195, SD = 2.761) over the control condition (M = 10.000, SD = 2.683) on the posttest.

**Table 4:** Post-instruction group statistics.

	Condition	*N*	Mean	Std. deviation	Std. error mean
Posttest scores	AI-DA	46	14.1957	2.76180	0.40721
	Control	46	10.0000	2.68328	0.39563

Table 5 nicely indicates that the AI-DA group significantly outperformed their peers in the control condition (*t* = 7.390, *df* = 90, *p* =0.001).

**Table 5:** Post-instruction independent samples test.

		Levene's test for equality of variances			*t*-test for equality of means					
		*F*	Sig.	*t*	df	Sig. (2-tailed)	Mean difference	Sth. error difference	95% confidence interval of the difference	
									Lower	Upper
Posttest scores	Equal variances assumed	0.004	0.947	7.390	90	0.000	4.19565	0.56775	3.06772	5.32358
	Equal variances not assumed			7.390	89.925	0.000	4.19565	0.56775	3.06771	5.32360

Table 6 demonstrates that if the effects of pretest scores are factored out, the posttest difference between the AI-DA and the control conditions still remains significant (*df* = 1, *F* = 53.980, *p* =0.001, Partial Eta Squared =0.378, Observed Power = 1.000).

**Table 6:** ANCOVA results.

Source	Type III sum of squares	df	Mean square	*F*	Sig.	Partial Eta squared	Noncent. parameter	Observed power
Corrected model	423.246	2	211.623	29.026	0.000	0.395	58.053	1.000
Intercept	1,124.756	1	1,124.756	154.272	0.000	0.634	154.272	1.000
Pretest scores	18.365	1	18.365	2.519	0.116	0.028	2.519	0.348
Condition	393.556	1	393.556	53.980	0.000	0.378	53.980	1.000
Error	648.874	89	7.291					
Total	14,537.000	92						
Corrected total	1,072.120	91						

## Discussion

This study investigated the role of AI-DA in shaping Chinese EFL learners' reading comprehension, academic buoyancy, and academic resilience. The results revealed three main findings. First, the qualitative analyses suggested that AI-DA fostered greater academic buoyancy, as learners reported being better able to “bounce back” from challenges, maintain motivation, and manage daily academic pressures. Second, the analyses indicated that AI-DA supported academic resilience, enabling learners to sustain effort, adapt to difficulties, and employ constructive strategies to cope with adverse academic experiences. Third, the quantitative analyses demonstrated that the AI-DA group significantly outperformed the control group in reading comprehension, even after controlling for pretest performance. Together, these findings suggest that AI-DA can enhance both linguistic outcomes and learners' psychological resources, addressing an important gap in previous research.

The study's findings on reading comprehension build on the growing evidence that DA, by situating assessment within the ZPD, offers learners scaffolded opportunities for growth beyond what is possible with static testing (Kushki and Nassaji, 2024; Vygotsky, 1987). While previous DA studies have highlighted improvements in areas such as vocabulary (Jeon, 2023) and pragmatics (Bashiri and Ebadi, 2025), the present findings extend this line of research by showing that AI-driven mediation also strengthens reading comprehension—a skill that is foundational for academic success (National Reading Panel et al., 2000; Snow, 2002; Anmarkrud and Bråten, 2009; Daugaard et al., 2017). This outcome resonates with research indicating that DA captures and fosters learners' potential in ways that static measures cannot (Dixon et al., 2023). Importantly, the integration of AI mediation helped overcome limitations traditionally associated with DA, including its time-intensive and resource-heavy nature (Lantolf and Poehner, 2004; Grigorenko and Sternberg, 1998). By employing ChatGPT-4o as a co-mediator, the study demonstrated how AI can deliver individualized, adaptive, and context-sensitive mediation at scale, aligning with calls for technology-mediated DA in language education (Poehner et al., 2017).

The findings related to academic buoyancy further illustrate the promise of AI-DA. Previous scholarship has established that buoyancy enables learners to cope effectively with daily academic challenges and is associated with improved performance, engagement, and well-being (Martin and Marsh, 2008; Kim, 2026; Collie et al., 2015; Putwain et al., 2022; Miller et al., 2013; Mohammad Hosseini et al., 2024). In line with this research, learners in the AI-DA condition described how the immediate, tailored scaffolding they received enhanced their confidence and helped them remain motivated when faced with difficulties. From an SCT perspective, this effect can be theoretically explained by the psychological extension of the ZPD: AI-mediated scaffolding provided learners with contingent support, allowing them to internalize coping strategies and self-regulatory behaviors that directly enhance buoyancy. Such results underscore the importance of incorporating positive psychology constructs into DA research, a direction that has been largely neglected in past work. By linking AI-assisted mediation to academic buoyancy, the study highlights how technology can not only strengthen language performance but also support learners' adaptability in managing everyday academic pressures.

Similarly, the results concerning academic resilience enrich the understanding of how AI-DA affects learners' broader psychological development. Resilience, defined as positive adaptation in the face of significant adversity (Masten, 2018; Reyes et al., 2018; Hartmann et al., 2020; Luthar et al., 2000; Luthans, 2002; Masten, 2014), has been recognized as a key predictor of academic achievement, mental health, and long-term success (Ang et al., 2021; Tarriño-Concejero et al., 2024; Hirvonen et al., 2020; Reich et al., 2020). The participants in the AI-DA condition reported that the contingent mediation they received helped them sustain effort, regulate stress, and adapt to challenges during the reading tasks. Through repeated AI-mediated interactions within the learners' ZPD, participants internalized strategies for managing setbacks and regulating emotional responses, demonstrating how SCT mechanisms underpin the development of resilience alongside cognitive gains. These findings extend prior DA research, which has mainly concentrated on cognitive outcomes (Jeon, 2023; Poehner and Wang, 2021; Kao, 2022; Chen et al., 2022), by showing that AI-assisted mediation can also foster the non-cognitive strengths necessary for learners to persist in demanding academic environments. This provides evidence that AI-DA can function as a dual-support mechanism, simultaneously advancing linguistic competence and psychological resilience.

In sum, the study the study contributes to the literature in three main ways. First, it confirms that AI-DA can substantially improve reading comprehension, supporting claims that DA provides a more accurate and growth-oriented measure of learning potential than static assessment (Kushki and Nassaji, 2024; Vygotsky, 1987; Dixon et al., 2023). Second, it extends DA research beyond cognitive gains to include psychological constructs such as buoyancy and resilience, thereby aligning language assessment with broader goals of learner well-being and adaptability (Martin and Marsh, 2008; Kim, 2026; Hartmann et al., 2020; Luthar et al., 2000; Luthans, 2002; Masten, 2014). Third, it responds to the call for scalable and sustainable approaches to DA (Lantolf and Poehner, 2004; Grigorenko and Sternberg, 1998; Poehner et al., 2017) by demonstrating how AI—in this case, ChatGPT-4o—can act as a co-mediator to deliver timely, individualized, and effective mediation in large group contexts. Collectively, these findings suggest that AI-DA holds considerable promise as a pedagogical and psychological resource, bridging a critical gap between traditional DA, technology-mediated learning, and positive psychology in L2 education.

The findings of this study carry important implications for both theory and pedagogy. Theoretically, the study advances the scope of DA by demonstrating how AI can function as a co-mediator within the learner's ZPD. This extends Vygotskian perspectives on mediation by showing that scaffolding need not be limited to human interaction but can also be effectively delivered through adaptive AI systems. By explicitly linking AI-mediated support to learners' internalization of coping, self-regulation, and adaptive strategies, the study models a mechanism by which SCT explains psychological adaptability as an extension of cognitive mediation. In doing so, the study also contributes to the growing body of research at the intersection of DA and positive psychology, establishing that AI-DA not only enhances cognitive outcomes such as reading comprehension but also fosters psychological resources like academic buoyancy and resilience. This dual contribution suggests that DA, when supported by AI, can be re-conceptualized as a multidimensional practice that simultaneously targets language development and learner well-being.

Pedagogically, the study provides insights for language teachers, curriculum developers, and policymakers. For teachers, the findings highlight the potential of integrating AI-based tools, such as ChatGPT-4o, to deliver individualized and immediate scaffolding that would otherwise be difficult to achieve in large classrooms. For example, teachers could assign reading tasks in which students first attempt comprehension questions independently and then receive AI-assisted hints or prompts tailored to their responses, allowing the teacher to monitor progress and intervene only when necessary. Similarly, AI can offer alternative explanations or examples for students struggling with specific vocabulary or grammar points, ensuring timely support without interrupting the entire class. Such tools can help learners navigate comprehension difficulties, maintain motivation, and develop adaptive strategies, thereby creating a more supportive and learner-centered classroom environment. For curriculum designers, the study underscores the importance of embedding AI-mediated scaffolding into instructional practices, particularly in reading-focused programs where learners may encounter persistent challenges. For policymakers, the study suggests that incorporating AI-assisted DA into language curricula could make assessment both more diagnostic and more developmental, aligning educational practices with broader goals of fostering resilience and adaptability in learners. Policymakers might consider funding AI platforms that provide real-time scaffolding reports for educators, establishing professional development programs to train teachers in AI-mediated DA, and creating guidelines for integrating AI into standardized language programs while maintaining ethical and pedagogical standards. Collectively, these implications indicate that AI-DA can serve as a transformative approach to language education, bridging the gap between theory and practice while addressing both academic and psychological dimensions of learning.

## Conclusion

This study set out to explore the interplay between AI-DA, academic buoyancy, academic resilience, and L2 reading comprehension through a concurrent mixed-methods design. The findings provided robust evidence that AI-DA, operationalized through ChatGPT-4o, significantly enhanced learners' reading comprehension and fostered positive psychological constructs such as buoyancy and resilience. Learners in the AI-DA condition not only outperformed their peers in reading comprehension but also reported greater capacity to cope with daily academic challenges and adapt to stressful situations. These outcomes highlight the promise of AI-DA as a powerful pedagogical approach that integrates instruction and assessment while also supporting learners' well-being.

The novelty of this study lies in its pioneering attempt to examine AI as a co-mediator in DA, a dimension that has received virtually no empirical attention apart from Jeon's earlier work. By demonstrating that AI systems can effectively scaffold learners within their ZPD, the study pushes the boundaries of both Vygotskian DA and positive psychology in L2 learning. In doing so, it advances the field by proposing that AI-mediated scaffolding can simultaneously target cognitive and affective domains, thereby offering a more holistic perspective on language learning. The integration of AI-DA with constructs like buoyancy and resilience also broadens the theoretical reach of DA, situating it at the intersection of language development and learner psychology.

Despite its contributions, the study is not without limitations. The research was conducted with a relatively small sample drawn from two intact classes at a single Chinese university, which limits the generalizability of the findings. Moreover, the intervention lasted 16 weeks, and longer-term effects of AI-DA on reading comprehension and psychological constructs remain uncertain. The reliance on self-reported data in the qualitative phase may also have introduced subjectivity, even though credibility and dependability measures were taken. Future research could extend these findings by involving larger and more diverse populations, examining different language skills beyond reading, and exploring longitudinal outcomes of AI-DA. Additionally, future studies might compare different AI systems or hybrid approaches where AI and human mediation are combined, thereby offering a more nuanced understanding of AI's role in education.

In conclusion, this study provides compelling evidence that AI-DA has the potential to transform language education by making assessment simultaneously diagnostic, instructional, and psychologically supportive. By situating AI as a legitimate co-mediator in learners' developmental processes, it not only advances DA theory but also paves the way for pedagogical innovations that balance academic achievement with resilience and well-being.

## Data Availability

The raw data supporting the conclusions of this article will be made available by the authors, without undue reservation.
